# Physicochemical Determinants of Bioactivity in Acacia Gum-Derived Silver Nanoparticles: Enhanced Selective Toxicity Toward MCF-7 Breast Cancer Cells

**DOI:** 10.3390/ijms27073106

**Published:** 2026-03-29

**Authors:** Haifa A. Alqahtani, Mohammed Al-Hariri

**Affiliations:** 1 Department of Biology, College of Science, Imam Abdulrahman Bin Faisal University, Dammam 31441, Saudi Arabia; 2 Department of Physiology, College of Medicine, Imam Abdulrahman Bin Faisal University, Dammam 31441, Saudi Arabia

**Keywords:** Gum Arabic, nanoparticles, breast cancer, oxidative stress

## Abstract

Silver nanoparticles synthesized using natural polysaccharides have received attention for their biocompatibility and potential selective anticancer activity. In this study, the physicochemical properties and biological activity of silver nanoparticles prepared using gums from *Acacia senegal* (ASS) and *Acacia seyal* (ASY) were compared. The gums were analyzed to determine their physicochemical characteristics and used as natural reducing and stabilizing agents in nanoparticle synthesis. The resulting nanoparticles were characterized using UV–visible spectroscopy, FTIR, dynamic light scattering, and zeta potential analysis. Their cytotoxicity was evaluated in MCF-7 breast cancer cells and HEK-293 normal cells using MTT assay, flow cytometry, and intracellular reactive oxygen species (ROS) measurement. Both gums showed properties consistent with Gum Arabic, with a higher protein content in ASS. ASS-derived nanoparticles were smaller and had greater colloidal stability. Both formulations reduced MCF-7 cell viability in a dose-dependent manner, with lower IC_50_ values observed for the ASS-based nanoparticles. Apoptosis induction was associated with increased ROS generation. Limited cytotoxicity toward HEK-293 cells resulted in favorable selectivity indices. Acacia gum–mediated silver nanoparticles demonstrate selective anticancer activity, and gum composition significantly influences nanoparticle stability and bioactivity, supporting their potential application in breast cancer nanotherapy.

## 1. Introduction

Breast cancer remains one of the most prevalent malignancies worldwide and a leading cause of cancer-related mortality among women in 157 of 185 countries. In 2022, approximately 2.3 million new cases and 670,000 deaths were reported globally [1].

Breast cancer is commonly classified into four major molecular subtypes based on the expression of estrogen receptors (ERs), progesterone receptors (PRs), and human epidermal growth factor receptor-2 (HER2). Certain subtypes, particularly triple-negative breast cancer, do not respond to hormonal therapies, highlighting the urgent need for alternative therapeutic strategies [2].

In the Kingdom of Saudi Arabia (KSA), 77,513 female breast cancer cases were recorded between 1990 and 2021, with the highest incidence observed in women aged 45–49 years, followed by those aged 40–44, 50–54, and 35–39 years [3]. Despite substantial advances in chemotherapy, radiotherapy, and hormonal therapy, treatment efficacy remains compromised by systemic toxicity, limited selectivity, and the emergence of multidrug resistance [4].

In vitro evaluation of anticancer agents usually requires the use of both malignant and non-malignant cell lines to determine not only the therapeutic efficacy but also the selectivity and safety profile of the tested compounds. In many preliminary cytotoxicity and nanotoxicology studies, non-tumorigenic human cell lines such as HEK293 are frequently used as a general model of normal cells due to their stable growth, reproducible behavior in culture, and well-characterized responses to oxidative stress and nanoparticle exposure [5,6]. These cells provide a reliable system for assessing whether newly synthesized nanomaterials exert selective toxicity toward cancer cells while maintaining acceptable biocompatibility with non-cancerous human cells [7,8]. Although tissue-specific normal cell lines may offer a more direct comparison, HEK293 cells remain widely used in early-stage screening studies to evaluate the general safety of nanoparticles before proceeding to more specialized cellular models [8].

Nanotechnology has emerged as a promising strategy in cancer therapy, enabling targeted drug delivery, improved bioavailability, and reduced off-target toxicity [9]. In particular, the green synthesis of nanoparticles using natural biopolymers offers an environmentally friendly and biocompatible alternative to conventional chemical synthesis routes [10].

Most existing studies focused on metallic silver nanoparticles (AgNPs); however, alternative silver-based nanomaterials such as silver phosphate (Ag_3_PO_4_) have recently attracted attention for their high surface reactivity, strong oxidative potential, and ability to generate reactive oxygen species (ROS), which may enhance their biological and anticancer activity compared with conventional AgNPs [11]. In addition, the presence of phosphate groups may influence particle stability, surface charge, and interaction with biological systems, potentially resulting in different cellular responses [12,13]. Despite these promising characteristics, the green synthesis of Ag_3_PO_4_ nanoparticles using natural gums remains limited.

Among natural polymers, plant-derived polysaccharides have attracted increasing attention as reducing and stabilizing agents [14]. Gum Arabic (GA), a natural, edible biopolymer gum obtained from the trunk exudations of *Acacia senegal* trees, is a complex polysaccharide composed mainly of arabinogalactan, glucuronic acid, and glycoproteins [15]. Owing to its abundance, biocompatibility, emulsifying capacity, and antioxidant properties, GA has been widely utilized in the food, pharmaceutical, and cosmetic industries and has been the subject of increasing interest in research on green nanoparticle synthesis [16].

GA exhibits a broad range of biological activities, including antimicrobial, anti-inflammatory, and prebiotic effects. Clinically and experimentally, it has been applied in the management of conditions such as metabolic disturbances, rheumatoid arthritis, renal ailments, sickle cell anemia, gastrointestinal disorders, and periodontal disease [15].

Two major commercial sources of GA are *Acacia senegal* var. senegal (ASS), commonly known as “hashab gum,” and *Acacia seyal* var. seyal (ASY), known as “talha gum.” These gums differ in color, texture, protein content, and physicochemical properties [17]. Although GA-based nanoparticles have shown promising cytotoxic activity against several cancer cell lines, including MCF-7 breast cancer cells, few studies have directly compared nanoparticles derived from different Acacia species [18].

Although GA-based and other plant gum-derived nanoparticles have demonstrated promising anticancer activity against several tumor cell lines, including breast cancer cells [19], important gaps remain in the literature. Most studies have utilized GA without specifying or systematically comparing the botanical source, despite well-established biochemical and physicochemical differences between ASS and ASY gums [20]. In addition, direct side-by-side evaluations of nanoparticles derived from these two species with respect to particle size, surface charge, colloidal stability, cytotoxic selectivity toward cancer versus normal cells, and apoptosis-related mechanisms are limited. Furthermore, the extent to which species-dependent variations in GA composition influence oxidative stress generation and downstream apoptotic pathways remains poorly understood [21].

Addressing these gaps is essential for the rational optimization of green nanoparticle synthesis and for identifying the most suitable natural polymer source for breast cancer nanotherapy. Therefore, this study was conducted to synthesize and characterize GA-coated Ag_3_PO_4_ nanoparticles derived from ASS and ASY and comparatively evaluate their physicochemical properties, cytotoxic selectivity, ROS generation, and induction of apoptosis in MCF-7 breast cancer cells.

## 2. Results

### 2.1. Physicochemical Properties of Gums

The physicochemical characteristics of the gum samples derived from ASS and ASY were determined using standard AOAC procedures. The analyses included assessments of moisture, ash, and nitrogen content (Table 1). Noticeable differences in protein and nitrogen levels were observed between the two gum types, with ASS exhibiting a markedly higher protein content compared to ASY. The pH values of all gum samples ranged from 4.81 to 4.85, attributed to the presence of free carboxyl groups of D-glucuronic acid and 4-O-methyl-D-glucuronic acid residues.

### 2.2. Synthesis and Visual Observation of Gum Arabic Nanoparticles

**Figure 1:**
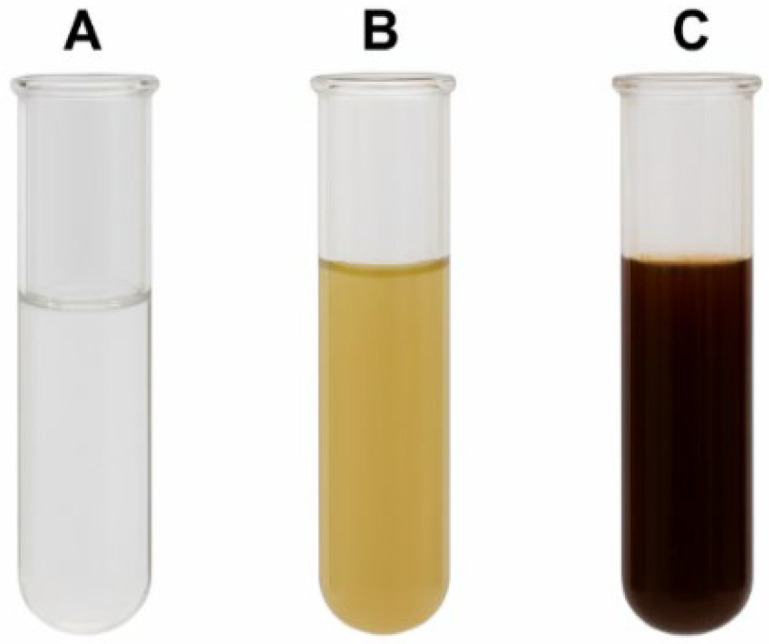
Color change during synthesis of GA-NPs: (**A**) AgNO_3_ solution; (**B**) reaction with ASY-GA extract; (**C**) reaction with ASS-GA extract after 30 min at 60 °C.

The formation of Gum Arabic-based silver nanoparticles (GA-NPs) was visually confirmed by the color transition of the reaction mixture. Upon mixing the GA extract with a silver nitrate solution and heating it at 60 °C for 30 min, the solution progressively changed from colorless to dark brown, indicating the successful reduction of Ag^+^ ions to silver nanoparticles. The ASS-GA-NP solution appeared slightly darker than that of ASY-GA-NPs, suggesting a higher yield and smaller particle size (Figure 1).

### 2.3. UV–Visible Spectroscopic Analysis

**Figure 2:**
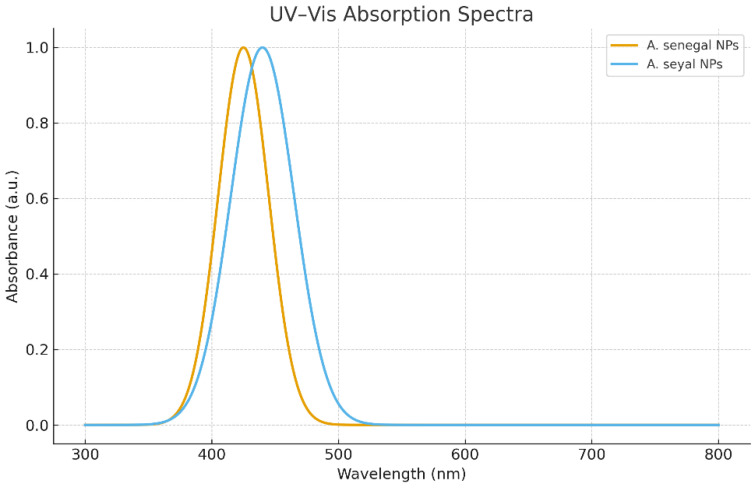
UV–visible spectra of ASS-GA-NPs and ASY-GA-NPs, showing SPR peaks around 425–440 nm.

The synthesis of GA-NPs was further confirmed by UV–visible spectroscopy. Distinct SPR peaks were observed between 420 and 450 nm, characteristic of silver nanoparticles (Figure 2). The ASS-GA-NPs exhibited a sharp and intense peak at approximately 425 nm, whereas the ASY-GA-NPs displayed a broader peak around 440 nm. The sharper SPR peak of the ASS-GA-NPs indicates a more uniform distribution and smaller nanoparticle size compared to the ASY-GA-NPs.

### 2.4. FTIR Spectroscopic Analysis

**Figure 3:**
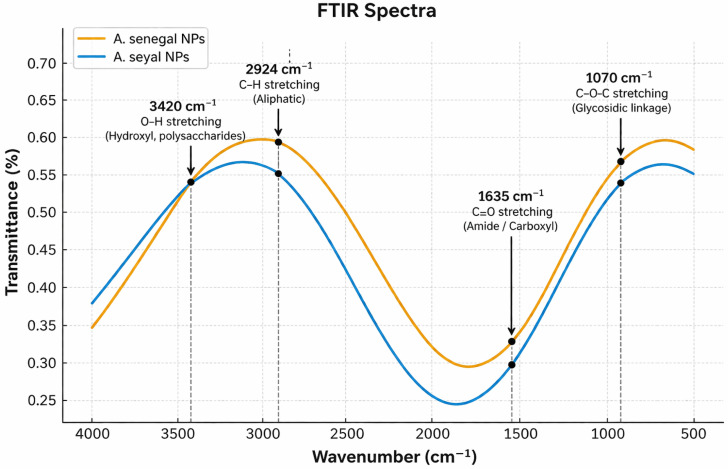
FTIR spectra of nanoparticles synthesized using *Acacia senegal* and *Acacia seyal* gum extracts showing characteristic bands corresponding to hydroxyl, aliphatic, carbonyl, and glycosidic functional groups involved in nanoparticle reduction and stabilization.

The FTIR spectra of both nanoparticle formulations confirmed the presence of functional groups responsible for nanoparticle reduction and stabilization (Table 2, Figure 3). Prominent absorption bands were observed at 3420 cm^−1^ (O–H stretching of polysaccharides), 2924 cm^−1^ (C–H stretching), 1635 cm^−1^ (C=O stretching of amide groups), and 1070 cm^−1^ (C–O–C stretching). These findings confirm the involvement of hydroxyl, carboxyl, and glycosidic moieties from the gum matrices acting as natural capping and stabilizing agents. Minor variations in peak intensities and positions between the ASS-GA-NPs and ASY-GA-NPs reflected compositional differences influencing their stability.

The FTIR spectra of the nanoparticles synthesized using ASS-GA-NPs and ASY-GA-NPs are shown in Figure 3, and the major absorption bands are summarized in Table 2. Both nanoparticle formulations exhibited characteristic peaks corresponding to functional groups commonly found in gum Arabic polysaccharides and proteinaceous components, indicating their role in nanoparticle reduction and stabilization.

A broad absorption band was observed at 3420 cm^−1^ for Sample A and 3415 cm^−1^ for Sample B, which corresponds to O–H stretching vibrations of hydroxyl groups present in polysaccharides. The peaks detected at 2924 cm^−1^ and 2920 cm^−1^ were assigned to aliphatic C–H stretching. In addition, bands located at 1635 cm^−1^ and 1628 cm^−1^ are attributed to C=O stretching of amide or carboxyl groups, suggesting the presence of protein residues associated with the gum matrix. The absorption bands observed at 1070 cm^−1^ and 1064 cm^−1^ correspond to C–O–C stretching vibrations of glycosidic linkages, confirming the polysaccharide structure of the stabilizing agents.

The presence of these functional groups indicates that the natural constituents of the gum extracts acted as reducing and capping agents during nanoparticle synthesis. Minor differences in peak position and intensity between ASS-GA-NPs and ASY-GA-NPs may reflect compositional variations between the two gum sources, which could contribute to differences in nanoparticle stability and physicochemical properties.

Slight differences between the present FTIR spectra and previously reported spectra of gum-stabilized silver nanoparticles may arise from variations in gum source, protein and polysaccharide composition, synthesis conditions, and FTIR measurement mode [Attenuated Total Reflectance (ATR) vs. potassium bromide (KBr)]. Such factors are known to influence peak position and intensity in polysaccharide-based nanoparticle systems.

### 2.5. Zeta Potential and Particle Size Distribution

DLS and zeta potential analyses revealed that the nanoparticles exhibited good colloidal stability (Table 3). The ASS-GA-NPs showed a surface charge of −32.6 mV compared to −25.8 mV for the ASY-GA-NPs. The average hydrodynamic diameters indicated smaller and more stable nanoparticles in the ASS-GA-NP formulation.

### 2.6. Cytotoxicity of GA-NPs on Breast Cancer Cell Lines (MTT Assay)

The cytotoxic activity of the ASS-GA-NPs and ASY-GA-NPs against MCF-7 breast cancer cells and HEK-293 normal cells was evaluated using the MTT assay after treatment with serial concentrations ranging from 6.25 to 100 µg/mL for 24, 48, and 72 h. This concentration range was selected to include doses below and above the calculated IC_50_ values in cancer cells while remaining within the non-toxic range for normal cells, facilitating accurate assessment of dose-dependent cytotoxicity and selectivity.

**Figure 4:**
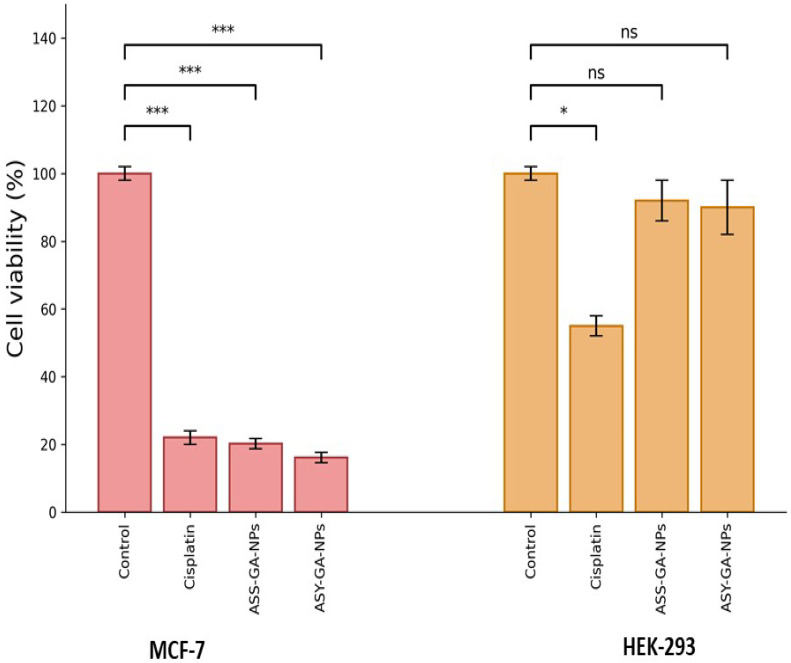
Cell viability assay showing the effects of ASS-GA-NPs and ASY-GA-NPs on MCF-7 breast cancer cells and HEK-293 normal cells. Cells were treated under the indicated conditions, and viability was expressed as a percentage relative to untreated control cells. Data are presented as mean ± SD of three independent experiments. Statistical significance was analyzed using one-way ANOVA followed by Tukey’s post hoc test. *** *p* < 0.001; * *p* < 0.05; ns: not significant; ASS: *Acacia senegal*; ASY: *Acacia seyal*; GA-NPs: Gum Arabic–based nanoparticles.

Figure 4 shows a representative comparison of cell viability at the selected concentration used for formulation with the positive control (cisplatin). At this concentration, both the ASS-GA-NPs and ASY-GA-NPs markedly reduced the viability of MCF-7 cells to 20.2% and 16.1%, respectively, whereas no significant reduction in viability was observed in HEK-293 cells under the same conditions.

The IC_50_ values calculated for the ASS-GA-NPs, ASY-GA-NPs, and cisplatin were 20.45, 20.05, and 20.10 µg/mL, respectively, while the CC_50_ values in HEK-293 cells were 200, 201, and 51.2 µg/mL, confirming that both nanoparticle formulations exhibited strong antiproliferative activity against cancer cells with considerably lower toxicity toward normal cells compared with the control (cisplatin), as illustrated in Figure 4.

**Figure 5:**
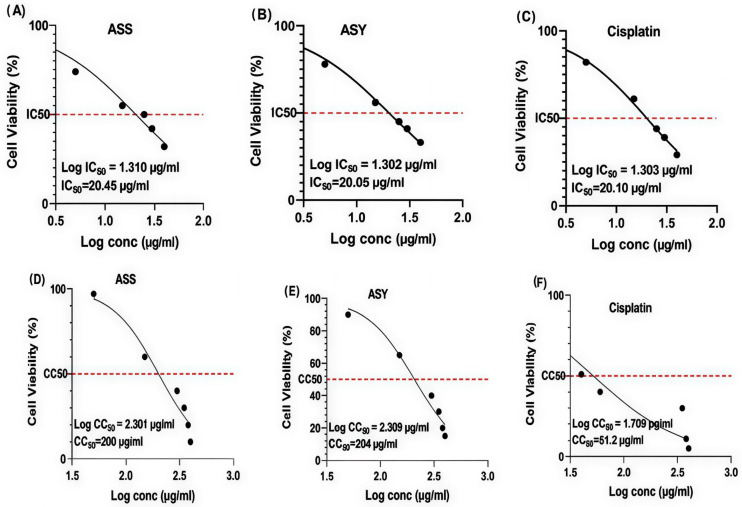
Evaluation of cytotoxicity of ASS, ASY, and cisplatin-based formulations on IC_50_ and CC_50_ values, indicating comparable anticancer potency but lower cytotoxicity of ASS and ASY toward normal cells compared with cisplatin. ASS: *Acacia senegal*; ASY: *Acacia seyal*; GA-NPs: Gum Arabic-based silver nanoparticles. (**A**–**C**) Determination of IC_50_ values in MCF-7 breast cancer cells treated with ASS-GA-NPs (**A**), ASY-GA-NPs (**B**), and cisplatin (**C**). (**D**–**F**) Determination of CC_50_ values in HEK-293 normal cells treated with ASS-GA-NPs (**D**), ASY-GA-NPs (**E**), and cisplatin (**F**). Cell viability (%) was plotted against the logarithm of concentration (µg/mL), and IC_50_ and CC_50_ values were calculated from nonlinear regression analysis of dose–response curves. Data represent the mean ± SD of three independent experiments.

In contrast, both nanoparticle formulations showed minimal cytotoxicity toward normal HEK-293 cells, with cell viabilities of 85.20% (ASS-GA-NPs) and 87.11% (ASY-GA-NPs) compared to 36.31% for cisplatin. The IC_50_ values calculated for the ASS-GA-NPs, ASY-GA-NPs, and cisplatin were 20.45, 20.05, and 20.10 µg/mL, respectively, whereas the CC_50_ values (200, 201, and 51.2 µg/mL) revealed that both nanoparticle formulations were considerably less toxic to normal cells (Figure 5A–F).

### 2.7. Morphological Changes

**Figure 6:**
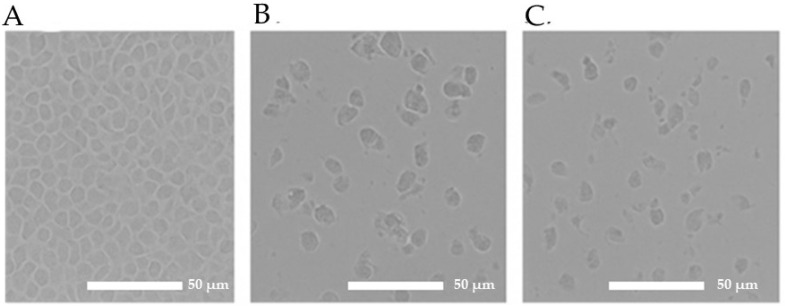
Morphological alterations in MCF-7 cells after 48 h of treatment with ASS-GA-NPs and ASY-GA-NPs at a concentration close to the calculated IC_50_ value. (**A**) Control cells, (**B**) ASS-GA-NP–treated cells, and (**C**) ASY-GA-NP–treated cells. Images were captured using an inverted microscope at 20× magnification. Scale bars = 50 µm. ASS: *Acacia senegal*; ASY: *Acacia seyal*; GA-NPs: Gum Arabic-based silver nanoparticles.

Morphological alterations in MCF-7 cells were evaluated after 48 h of treatment with the ASS-GA-NPs and ASY-GA-NPs at concentrations close to their calculated IC_50_ values. As shown in Figure 6, the untreated control cells exhibited normal epithelial morphology, with intact cell membranes and dense monolayer growth. In contrast, the treated cells showed marked cytotoxic features, including cell shrinkage, rounding, loss of adhesion, and reduced cell density. These morphological changes were consistent with the MTT assay results and confirm the antiproliferative activity of both nanoparticle formulations against MCF-7 breast cancer cells.

### 2.8. Apoptosis Induction Analysis

Flow cytometric analysis using Annexin V–FITC/PI staining was carried out to evaluate apoptosis induction in both MCF-7 breast cancer cells and HEK-293 normal kidney cells after treatment with GA-based nanoparticles. Cells were exposed to ASS-GA-NPs and ASY-GA-NPs at concentrations of 10, 25, 50, and 100 µg/mL for 24 h, based on the IC_50_ values obtained from the MTT assay, while cisplatin (10 µg/mL, 24 h) was used as a positive control.

**Figure 7:**
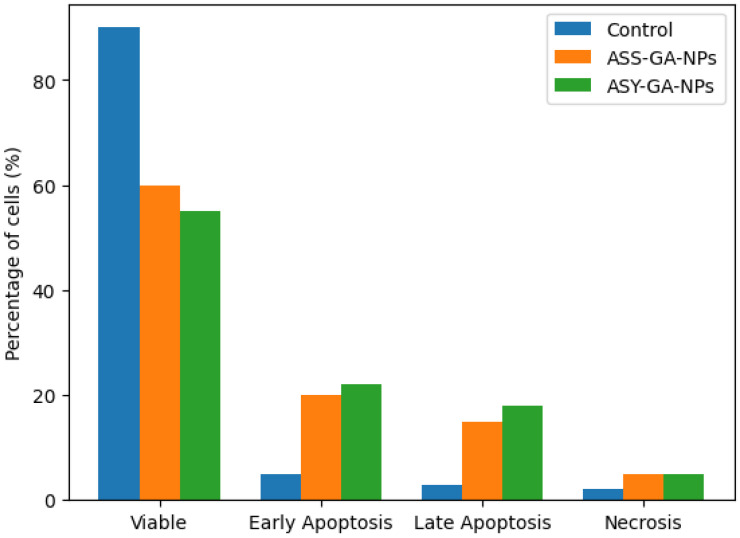
Apoptosis analysis in MCF-7 cells after 48 h treatment with ASS-GA-NPs and ASY-GA-NPs at a concentration close to the calculated IC_50_ value, evaluated by flow cytometry using Annexin V–FITC/PI staining. Percentages of viable, early apoptotic, late apoptotic, and necrotic cells are shown for control and treated groups.

**Figure 8:**
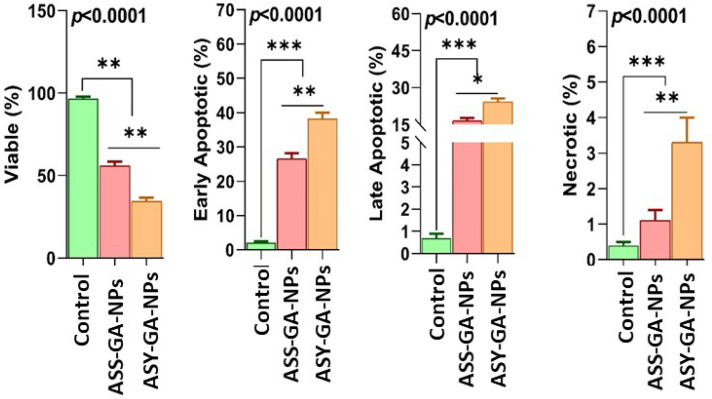
Effects of ASS-GA-NP and ASY-GA-NP treatments on cell viability and apoptotic profile of MCF-7 cells after 24 h of exposure at concentrations based on IC_50_ values. Percentages of viable, early apoptotic, late apoptotic, and necrotic cells were determined by flow cytometry using Annexin V–FITC/PI staining. Data are presented as mean ± SD of three independent experiments. Statistical analysis was performed using one-way ANOVA followed by Tukey’s post hoc test. * *p* < 0.05, ** *p* < 0.01, *** *p* < 0.001, and *p* < 0.0001 indicate significant differences compared with control group. ASS: *Acacia senegal*; ASY: *Acacia seyal*; GA-NPs: Gum Arabic-based silver nanoparticles.

**Figure 9:**
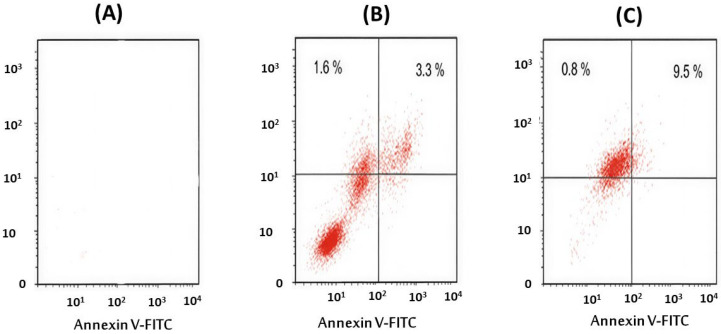
Flow cytometry plots of Annexin V–FITC/PI staining showing apoptosis in MCF-7 cells treated with (**A**) control, (**B**) ASY-GA-NPs, and (**C**) ASS-GA-NPs. ASS: *Acacia senegal*; ASY: *Acacia seyal*; GA-NPs: Gum Arabic-based silver nanoparticles.

In the MCF-7 cells, flow cytometry revealed a clear increase in apoptotic cell populations following nanoparticle treatment compared with the untreated control cells (Figure 7, Figure 8 and Figure 9). As illustrated in Figure 7, treatment with the GA-NPs resulted in a marked reduction in the percentage of viable cells, accompanied by a significant increase in early and late apoptotic fractions. The total apoptotic population increased from 43.1% in the ASY-GA-NP–treated cells to 62.4% in the ASS-GA-NP–treated cells (*p* < 0.05). The ASS-GA-NPs had the strongest effect, showing the lowest viable cell percentage and the highest proportions of early apoptosis, late apoptosis, and necrosis (*p* < 0.0001), with an activity comparable to that observed in the cisplatin-treated group (Figure 7, Figure 8 and Figure 9).

In contrast, the HEK-293 cells showed considerably lower apoptotic responses under the same treatment conditions. Only slight increases in apoptotic fractions were detected even at the highest tested concentration, indicating that the nanoparticles exerted limited cytotoxic effects on normal cells. These findings confirm that GA-NPs, particularly ASS-GA-NPs, selectively induce apoptosis in MCF-7 cancer cells while maintaining low toxicity toward HEK-293 cells, demonstrating the selective pro-apoptotic and anticancer potential of the synthesized nanoparticles (Figure 7, Figure 8 and Figure 9).

### 2.9. Reactive Oxygen Species (ROS) Generation

Intracellular reactive oxygen species levels were evaluated using the DCFH-DA fluorescence assay. Treatment with the GA-NPs resulted in a significant increase in ROS production compared with the untreated control cells. The increase in fluorescence intensity was more pronounced in the cells treated with ASS-GA-NPs than in those treated with ASY-GA-NPs, indicating a higher level of oxidative stress in the ASS-GA-NP group.

**Figure 10:**
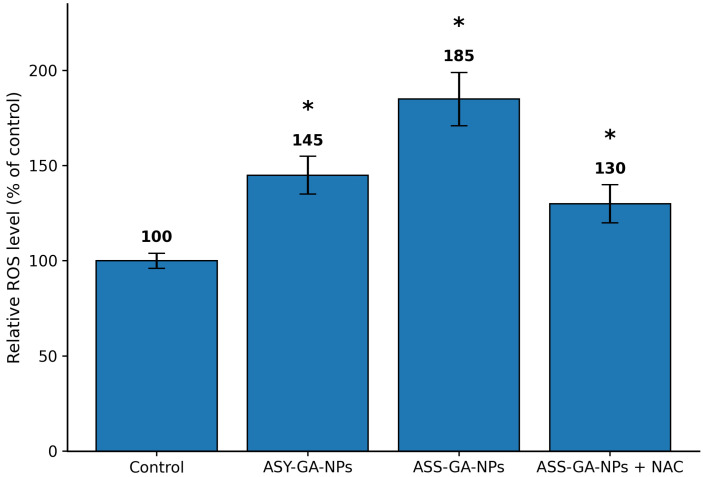
Intracellular reactive oxygen species (ROS) generation in MCF-7 cells after treatment with ASS-GA-NPs and ASY-GA-NPs, measured using the DCFH-DA fluorescence assay. Cells were exposed to nanoparticles at concentrations based on the IC_50_ values for 24 h. Treatment with GA-NPs significantly increased ROS production compared with untreated control cells, with ASS-GA-NPs inducing a higher level of oxidative stress than ASY-GA-NPs. Pretreatment with the antioxidant N-acetylcysteine (NAC) markedly reduced ROS generation, confirming the involvement of oxidative stress in GA-NP-induced cytotoxicity. Data are presented as mean ± SD of three independent experiments. Statistical analysis was performed using one-way ANOVA followed by Tukey’s post hoc test. * *p* < 0.05 vs. control. ROS: reactive oxygen species; ASS: *Acacia senegal*; ASY: *Acacia seyal*; GA-NPs: Gum Arabic-based silver nanoparticles; NAC: N-acetylcysteine.

Furthermore, pretreatment with the antioxidant N-acetylcysteine markedly attenuated ROS generation and partially restored cell viability, supporting the involvement of oxidative stress in GA-NP–induced cytotoxicity. These findings suggest that enhanced ROS production may contribute to mitochondrial damage and the subsequent induction of apoptotic cell death (Figure 10).

## 3. Discussion

This study demonstrates that silver nanoparticles synthesized using gums from ASS and ASY possess significant in vitro anticancer activity against MCF-7 breast cancer cells, primarily via ROS-associated apoptotic pathways. ASS-GA-NPs in particular had superior potency, likely due to their smaller size, higher negative surface charge, and the distinct biochemical composition of the parent gum. Both formulations exhibited favorable selectivity toward cancer cells over normal HEK-293 cells, underscoring their potential as safer alternatives or adjuncts to conventional chemotherapeutic agents [22].

The physicochemical characterization of ASS and ASY gums in this study aligns with previously reported specifications for Gum Arabic, particularly regarding moisture content, ash, and the general compositional profile of these exudates. The ASS gum showed higher protein and nitrogen contents relative to the ASY gum, consistent with earlier work indicating that ASS gums typically possess superior surface and emulsifying properties compared with ASY gums. The slightly acidic pH values (4.81–4.85) observed for all gum samples can be attributed to the presence of glucuronic and 4-O-methyl-D-glucuronic acid residues in the polysaccharide backbone, which is in agreement with previous descriptions of Gum Arabic acidity [23].

Green synthesis of silver nanoparticles using these gums produced stable GA-NPs with distinct optical and surface characteristics. The ASS-GA-NPs displayed a sharper surface plasmon resonance band and more negative zeta potential than ASY-GA-NPs, indicating a smaller particle size and better colloidal stability. Such physicochemical features are known to critically influence cellular uptake, intracellular localization, and ultimately the biological activity of nanomaterials. The natural macromolecular structure of Gum Arabic, including its arabinogalactan–protein fractions, likely contributes to efficient reduction and capping of silver ions, thereby enhancing nanoparticle stability and bioactivity [24]. However, differences in the chemical composition of ASS and ASY gums may affect nanoparticle formation efficiency and consequently influence particle size, zeta potential, and actual nanoparticle yield. In the present study, the concentration was estimated based on precursor amounts under identical synthesis conditions, and therefore, small variations in nanoparticle yield cannot be excluded.

The cytotoxicity results demonstrated that both ASS-GA-NPs and ASY-GA-NPs significantly reduced MCF-7 breast cancer cell viability in a dose-dependent manner, with the ASS-GA-NPs having a lower IC_50_ and thus higher potency. This pattern is consistent with other reports on plant-mediated silver nanoparticles, where small, highly charged particles effectively induce cancer cell death through oxidative and mitochondrial stress pathways. In several green AgNP systems tested against MCF-7 cells, comparable IC_50_ ranges and ROS-driven apoptotic responses have been reported, suggesting that the activity observed here is within a pharmacologically relevant window. Flow cytometry confirmed apoptosis as the predominant mode of cell death, reflected by increased Annexin V-positive populations and a marked decline in viable cells following treatment. Concomitant elevation in intracellular ROS levels further supports a ROS-mediated apoptotic mechanism, which is a recognized route for many phytochemical- and nanoparticle-based anticancer agents [25].

This study demonstrated that GA-NP treatment significantly increased intracellular ROS production, particularly in cells treated with ASS-GA-NPs, indicating enhanced oxidative stress. Excessive ROS generation is a well-known mechanism of nanoparticle-induced cytotoxicity, as it disrupts cellular redox balance, leading to mitochondrial dysfunction and the activation of apoptotic pathways [26]. The reduction in ROS levels after pretreatment with N-acetylcysteine further supports the involvement of oxidative stress in GA-NP–induced cell death, since this antioxidant is known to restore intracellular glutathione and protect against free-radical-mediated damage [27]. Similar ROS-dependent apoptotic effects have been reported for silver and biopolymer-mediated nanoparticles in cancer cells, where increased oxidative stress triggers mitochondrial damage and caspase-mediated apoptosis [28]. The greater ROS production observed with ASS-GA-NPs may be related to differences in nanoparticle size and surface properties, which can influence cellular uptake and redox activity.

FTIR analysis revealed characteristic functional groups (hydroxyl, carbonyl, and glycosidic linkages) in the GA-NPs, indicating that gum-derived polysaccharides and associated biomolecules function as both reducing and stabilizing agents. These moieties not only participate in nanoparticle formation but may also retain intrinsic bioactivity, thereby contributing to the overall anticancer effect. Similar functional signatures have been associated with enhanced redox activity and pro-apoptotic signaling in other plant-derived nano constructs [24].

Importantly, both ASS and ASY-based systems showed preferential toxicity toward MCF-7 cells while exerting limited cytotoxic effects on normal HEK-293 cells, as reflected by their SI values. The SI is a key parameter in early anticancer screening, and values below 2 are generally interpreted as indicating poor selectivity and general toxicity, whereas higher values suggest more favorable therapeutic windows. In this context, the SI values obtained for GA-NPs, particularly for the ASY formulations, are encouraging and indicate meaningful differentiation between tumor and normal cells [29].

The phytochemical composition of ASS and ASY gums, including their content of flavonoids and related phenolics, may further explain their selective anticancer activity. Quercetin, one of the best-characterized dietary flavonols, has been extensively documented to induce apoptosis, modulate ROS levels, and inhibit proliferation in multiple cancer cell types with relatively limited toxicity to non-malignant cells. A higher total flavonoid content reported for ASY in some studies may account for its strong antiproliferative effects, complementing the nanoparticle-mediated mechanisms observed in this study. The interplay between metal-based nanostructures and flavonoid-rich organic matrices could lead to synergistic amplification of oxidative and apoptotic signaling in cancer cells [30].

One limitation of the current study is the absence of detailed phytochemical and monosaccharide characterizations of the ASS and ASY gum samples. In particular, the quantification of total phenolic and flavonoid contents and the determination of key sugar constituents, including D-glucuronic acid, 4-O-methyl-D-glucuronic acid, galactose, arabinose, and rhamnose, were not undertaken. These constituents are known to play an important role in defining the structural and functional properties of GA and may contribute to variations in nanoparticle synthesis, stability, and biological activity. Therefore, future studies should include comprehensive compositional profiling to clarify the extent to which differences in gum composition influence the physicochemical characteristics and anticancer effects of the resulting nanoparticles.

## 4. Materials and Methods

### 4.1. Plant Extraction and Sample Preparation

GA samples obtained from ASS and ASY were provided by the Natural Gums Research Centre of the Sudan University of Science and Technology, Khartoum, Sudan (season 2023). The products were also available at the local Dammam Market, Dammam, Eastern Province, Saudi Arabia. Saudi Arabia [31]. The samples were air-dried for 24 h, ground into a fine powder, and sieved through a 100 µm mesh. For aqueous extraction, 10 g of each powder was mixed with 100 mL of double-distilled water and stirred at 60 °C for 2 h. The mixtures were allowed to stand at room temperature for 48 h to ensure complete extraction. The resulting solutions were filtered using Whatman (Cytiva, Marlborough, MA, USA) No. 1 filter paper (20–24 µm pore size) and stored at 4 °C for use as stabilizing and reducing agents in subsequent nanoparticle synthesis [32].

**Figure 11:**
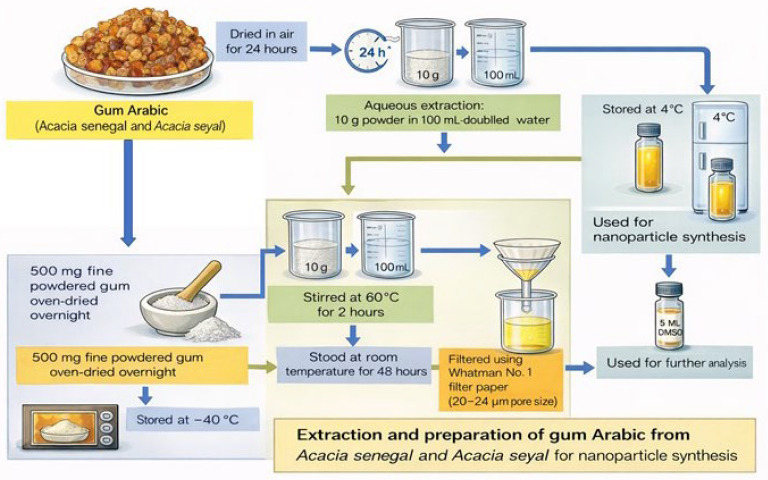
Schematic diagram of plant extraction and sample preparation.

Additionally, 500 mg of each powdered gum sample was oven-dried overnight, dissolved in 5 mL of dimethyl sulfoxide (DMSO; Sigma-Aldrich, St. Louis, MO, USA), and preserved at –40 °C for further analyses (Figure 11).

### 4.2. Physicochemical Characterization of Gum Arabic

The physicochemical parameters, including moisture, ash, and nitrogen content, of the ASS and ASY gums were determined in accordance with the standard methods of the Association of Official Analytical Chemists (AOAC) [33].

### 4.3. Synthesis of Gum Arabic-Coated Ag_3_PO_4_ Nanoparticles (NPs)

The synthesis conditions for the *Acacia senegal* (ASS-GA-NPs) and *Acacia seyal* (ASY-GA-NPs) Gum Arabic–mediated nanoformulations were optimized based on our previously reported method with minor modifications [34]. Briefly, aqueous solutions of Gum Arabic obtained from A. senegal and A. seyal were prepared by dissolving the purified gum powder in deionized water under continuous magnetic stirring at room temperature until a clear and homogeneous solution was formed. This solution was used as a natural stabilizing and capping agent during nanoparticle synthesis.

For the preparation of nanoparticles, 0.5 g of silver nitrate (AgNO_3_;; Sigma-Aldrich, St. Louis, MO, USA) was dissolved in 30 mL of deionized water and mixed with the corresponding Gum Arabic solution under continuous stirring, followed by sonication for 5 min to ensure uniform dispersion. Subsequently, 0.3 g of disodium hydrogen phosphate (Na_2_HPO_4_; Sigma-Aldrich, St. Louis, MO, USA), dissolved in 10 mL of deionized water, was added dropwise to the reaction mixture under continuous ultrasonication for 40 min to promote the formation of Gum Arabic-coated Ag_3_PO_4_ nanoparticles. The presence of Gum Arabic in the reaction medium provides hydroxyl and carboxyl functional groups that facilitate nucleation, stabilization, and the prevention of nanoparticle aggregation during the synthesis process.

Successful nanoparticle formation was indicated by the appearance of a brownish-yellow coloration in the reaction mixture. The obtained colloidal suspension was centrifuged at 10,000 rpm for 20 min, washed twice with distilled water followed by ethanol to remove unreacted components, and then dried overnight at 60 °C to obtain a dry nanoparticle powder. The nanoformulations synthesized using A. senegal and A. seyal gums were designated as ASS-GA-NPs and ASY-GA-NPs, respectively [35].

### 4.4. Visual Observation of Nanoparticle Formation

The concentration of the synthesized nanoparticles was estimated based on the initial amount of silver nitrate used during synthesis and the final volume of the nanoparticle suspension after centrifugation and washing. Identical synthesis conditions were applied for both gum types to enable direct comparison between preparations. The formation of nanoparticles was verified by a distinct color change in the reaction mixture when the gum extract was mixed with the silver nitrate solution and heated at 60 °C for 30 min [36].

### 4.5. UV–Visible Spectroscopy

The optical characteristics of the synthesized ASS-GA-NPs and ASY-GA-NPs were analyzed using a UV–visible spectrophotometer (UV-1800, Shimadzu Corporation, Kyoto, Japan). The surface plasmon resonance (SPR) bands observed between 420 and 450 nm confirmed the formation of silver phosphate nanoparticles [37].

### 4.6. Fourier Transform Infrared Spectroscopy (FTIR)

Functional groups present in the plant extracts and nanoparticles were identified using a Fourier-transform infrared (FTIR) spectrometer (PerkinElmer Inc., Waltham, MA, USA) equipped with an attenuated total reflectance (ATR) accessory. The background spectrum was recorded using a clean ATR crystal, followed by the placement of a small sample amount on the crystal. Spectra were collected over a range of 500–4000 cm^−1^ at a resolution of 4 cm^−1^, with 32 scans per sample [38]. 

### 4.7. Zeta Potential and Particle Size Analysis

The average hydrodynamic diameter, polydispersity index (PDI), and zeta potential of the synthesized nanoparticles were determined using Dynamic Light Scattering (DLS) with a Zetasizer Nano ZS instrument (Malvern Panalytical Ltd., Malvern, UK). Measurements were carried out at 25 °C after appropriate dilution of the nanoparticle suspension with deionized water to avoid multiple scattering effects. Particle size distribution obtained from the DLS analysis was used to calculate the PD, which reflects the uniformity of the nanoparticle population. The PDI values were derived from the cumulant analysis of the autocorrelation function according to the standard relation

PDI = (σ/d)^2^

where σ represents the standard deviation of the particle size distribution, and d is the mean hydrodynamic diameter. Lower PDI values indicate a more homogeneous nanoparticle dispersion, whereas higher values suggest a broader size distribution.

Zeta potential measurements were performed using the same instrument based on electrophoretic light scattering to evaluate the surface charge and colloidal stability of the nanoparticles. Higher absolute zeta potential values indicate greater electrostatic repulsion between particles and improved stability of the colloidal system [39].

### 4.8. Cell Lines and Cell Culture

Human breast adenocarcinoma cells (MCF-7, ER-positive; ATCC^®^ HTB-22™) and human embryonic kidney cells (HEK-293; ATCC^®^ CRL-1573™) were obtained from the American Type Culture Collection (ATCC, Manassas, VA, USA). All in vitro experiments were conducted at the Pharmaceutical Laboratory, College of Medicine, King Faisal University, Saudi Arabia. Cells were cultured according to ATCC recommendations for continuous mammalian cell lines to ensure reproducibility and optimal growth conditions.

Cells were maintained in Dulbecco’s Modified Eagle Medium (DMEM, high glucose) supplemented with 10% fetal bovine serum (FBS), L-glutamine, sodium selenite, and 1% penicillin–streptomycin (100 U/mL penicillin and 100 µg/mL streptomycin). DMEM, FBS, L-glutamine, penicillin–streptomycin, trypsin–EDTA, and phosphate-buffered saline (PBS) were purchased from Gibco, Thermo Fisher Scientific (Waltham, MA, USA). Cell cultures were incubated at 37 °C in a humidified atmosphere containing 5% CO_2_ and were subcultured at approximately 70–80% confluence using a trypsin–EDTA solution. The culture medium was replaced every 2–3 days, and only cells in the logarithmic growth phase were used for experiments.

For cytotoxicity assessment, cells were treated with silver nanoparticles synthesized using *Acacia senegal* gum (ASS-AgNPs) and *Acacia seyal* gum (ASY-AgNPs) at concentrations of 6.25, 12.5, 25, 50, and 100 µg/mL for 24 h. Following treatment, cell viability was evaluated using the MTT assay, as previously described [40].

The cytotoxic effects of the synthesized GA-NPs were determined by measuring the reduction of MTT to formazan crystals, which reflects mitochondrial metabolic activity in viable cells [41].

### 4.9. Cell Viability Assay (MTT Assay)

Cell viability was assessed using the colorimetric MTT assay, following previously established protocols with minor modifications [42]. Briefly, cells were seeded into 96-well plates at a density of 1 × 10^4^ cells per well in 100 µL of complete culture medium and allowed to attach overnight under standard incubation conditions. After cell attachment, the medium was replaced with fresh medium containing serial concentrations of the tested extracts or nanoparticles. Cells were treated in triplicate with concentrations ranging from 6.25 to 100 µg/mL, while untreated cells cultured in complete medium served as the control group. The selected concentration range was based on preliminary optimization experiments, previously published studies evaluating the cytotoxic activity of silver nanoparticles in mammalian cell lines, and to cover concentrations around the expected IC_50_ values in cancer cells while remaining below the CC_50_ values in normal cells to enable accurate determination of selectivity. Cisplatin was used as a positive control and tested under the same experimental conditions.

After 24, 48, and 72 h of incubation at 37 °C in a humidified atmosphere containing 5% CO_2_, 10 µL of MTT solution (5 mg/mL) was added to each well, and plates were further incubated for 4 h to allow the formation of formazan crystals. The supernatant was then carefully removed, and 100 µL of dimethyl sulfoxide (DMSO) was added to each well to dissolve the formed crystals. After 24, 48, and 72 h of incubation at 37 °C in a humidified atmosphere containing 5% CO_2_, 10 µL of MTT solution (5 mg/mL) was added to each well, and the plates were further incubated for 4 h to allow the formation of formazan crystals. The supernatant was then carefully removed, and 100 µL of dimethyl sulfoxide (DMSO) was added to each well to dissolve the formed crystals. After gentle shaking for 5 min, absorbance was measured at 570 nm using a microplate reader (TECAN Group Ltd., Männedorf, Switzerland), with a reference wavelength of 630 nm. Cell viability was expressed as a percentage relative to untreated control cells [43].

### 4.10. Selectivity Index (SI)

The selectivity of the tested formulations toward cancer cells was evaluated by calculating the selectivity index (SI) based on their half-maximal inhibitory concentration (IC_50_) in MCF-7 breast cancer cells and half-maximal cytotoxic concentration (CC_50_) in non-cancerous HEK-293 cells, as described previously [44]. The SI was calculated using the following equation:

SI = CC50 in a non-cancer cell line HEK 293 cells/IC50 in cancer cell line MCF-7 × 100

Compounds exhibiting higher SI values were considered more selective and were prioritized for subsequent optimization and mechanistic studies.

### 4.11. Morphological Assessment

Morphological alterations in treated and untreated cells were examined using an inverted phase-contrast microscope (Olympus IX71, Olympus Corporation, Tokyo, Japan). MCF-7 cells were seeded in culture plates, allowed to attach overnight, and then treated with ASS-GA-NPs and ASY-GA-NPs at a concentration close to the calculated IC_50_ value for 48 h under standard incubation conditions. Untreated cells served as the control group. After treatment, the cells were examined for characteristic features associated with cytotoxicity and apoptosis, including cell shrinkage, rounding, loss of adherence, membrane blebbing, and cellular fragmentation [45]. Representative photomicrographs were captured at 20× magnification, and scale bars were added using the microscope calibration software to enable accurate visualization of cellular morphology.

### 4.12. Apoptosis Analysis by Annexin V–FITC/PI Staining

Apoptotic cell death was quantified using an Annexin V–FITC/propidium iodide (PI) apoptosis detection kit (BD Biosciences, San Jose, CA, USA). Cells were treated with GA-NP formulations at their respective IC_50_ concentrations for 24 h, harvested, and washed twice with cold phosphate-buffered saline (PBS). Cells were then resuspended in binding buffer and stained with Annexin V–FITC and PI for 15 min in the dark, according to the manufacturer’s instructions.

Samples were analyzed using flow cytometry (BD FACSCalibur, BD Biosciences, San Jose, CA, USA), and the percentages of viable, early apoptotic, late apoptotic, and necrotic cells were quantified [46].

### 4.13. Reactive Oxygen Species (ROS) Assay

Intracellular ROS generation was assessed using 2′,7′-dichlorofluorescin diacetate (DCFH-DA). Cells treated with ASS-GA-NPs and ASY-GA-NPs were incubated with 10 µM DCFH-DA for 30 min at 37 °C in the dark. Fluorescence intensity was measured at excitation/emission wavelengths of 485/528 nm using a microplate reader. ROS production was expressed as relative fluorescence intensity compared with untreated control cells [47].

### 4.14. Statistical Analysis

Statistical analyses were performed using GraphPad Prism software (version 5 or higher). Data normality was assessed using the Shapiro–Wilk test. Normally distributed data are presented as mean ± SD. Differences among multiple groups were analyzed by one-way ANOVA followed by Tukey’s post hoc test, while non-parametric data were analyzed using Dunn’s multiple comparison test. IC_50_ and CC_50_ values were calculated using nonlinear regression based on dose–response curves. A *p*-value < 0.05 was considered statistically significant.

## 5. Conclusions

Overall, these findings highlight that both the botanical source and physicochemical profile of the gum significantly shape the biological performance of the resulting nanoparticles. Optimizing parameters such as particle size, surface charge, and organic corona composition may further enhance their anticancer efficacy and selectivity. Future work should therefore focus on the systematic modulation of synthesis conditions, detailed pathway analysis (including caspase activation and mitochondrial membrane potential), and in vivo validation in relevant breast cancer models.

## Figures and Tables

**Table 1:** Physicochemical properties of the gum samples.

Gum	Moisture%	Ash%	Nitrogen%	Protein%	pH
ASS	9.73	3.38	0.412	2.141	4.85
ASY	8.52	3.16	0.235	1.431	4.81

ASS: *Acacia senegal*; ASY: *Acacia seyal*.

**Table 2:** Major FTIR absorption bands and functional group assignments of ASS-GA-NPs and ASY-GA-NPs.

Vibrational Mode	Reference Range (cm^−1^)	Sample A (cm^−1^)	Sample B (cm^−1^)	Functional Group Assignment
O–H stretching	3400–3450	3420	3415	Hydroxyl (–OH) groups
C–H stretching	2900–2950	2924	2920	Aliphatic C–H bonds
C=O stretching	1620–1650	1635	1628	Amide/carboxyl groups
C–O–C stretching	1050–1100	1070	1064	Glycosidic linkages

**Table 3:** Particle size and zeta potential of ASS-GA-NPs and ASY-GA-NPs.

Parameter	ASS-GA-NPs	ASY-GA-NPs
Hydrodynamic size (DLS)	36.4 ± 3.2 nm	45.1 ± 4.7 nm
Zeta potential (mV)	−32.6 ± 1.5	−25.8 ± 1.3
Polydispersity index (PDI)	0.268	0.354

ASS: *Acacia senegal*; ASY: *Acacia seyal*; GA-NPs: Gum Arabic-based silver nanoparticles.

## Data Availability

The raw data supporting the conclusions of this article will be made available by the authors on request.

## References

[1] Sung H., Ferlay J., Siegel R.L., Laversanne M., Soerjomataram I., Jemal A., Bray F. (2021). Global Cancer Statistics 2020: GLOBOCAN Estimates of Incidence and Mortality Worldwide for 36 Cancers in 185 Countries. CA Cancer J. Clin..

[2] Orrantia-Borunda E., Anchondo-Nuñez P., Acuña-Aguilar L.E., Gómez-Valles F.O., Ramírez-Valdespino C.A., Mayrovitz H.N. (2022). Subtypes of Breast Cancer. Breast Cancer.

[3] Al Zomia A.S., Al Zehefa I.A.M., Lahiq L.A., Mirdad M.T., Alshahrani A.S., Alshahrani T., Almahfuth N.N., Mirdad M.T., Alqarni A.A., Alshareef N.M. (2024). Tracking the Epidemiological Trends of Female Breast Cancer in Saudi Arabia since 1990 and Forecasting Future Statistics Using Global Burden of Disease Data, Time-Series Analysis. BMC Public Health.

[4] Zafar A., Khatoon S., Khan M.J., Abu J., Naeem A. (2025). Advancements and Limitations in Traditional Anti-Cancer Therapies: A Comprehensive Review of Surgery, Chemotherapy, Radiation Therapy, and Hormonal Therapy. Discov. Oncol..

[5] Smesnik G., Virgolini N., Toth M., Dürauer A., Borth N. (2026). Comparative Analysis of HEK293 Genomic Variability. Biotechnol. Bioeng..

[6] Shen C., Gu M., Song C., Miao L., Hu L., Liang D., Zheng C. (2008). The Tumorigenicity Diversification in Human Embryonic Kidney 293 Cell Line Cultured In Vitro. Biologicals.

[7] Jeyarani S., Vinita N.M., Puja P., Senthamilselvi S., Devan U., Velangani A.J., Biruntha M., Pugazhendhi A., Kumar P. (2020). Biomimetic Gold Nanoparticles for Its Cytotoxicity and Biocompatibility Evidenced by Fluorescence-Based Assays in Cancer (MDA-MB-231) and Non-Cancerous (HEK-293) Cells. J. Photochem. Photobiol. B.

[8] Liu X., Shan K., Shao X., Shi X., He Y., Liu Z., Jacob J.A., Deng L. (2021). Nanotoxic Effects of Silver Nanoparticles on Normal HEK-293 Cells in Comparison to Cancerous HeLa Cell Line. Int. J. Nanomed..

[9] Razavi Z., Mottaghi A., Dmitrieva L., Fallahianshafiei S., Olfat E., Ahmadi N. (2026). Precision Nanotechnology: Revolutionizing Therapeutic Strategies Against Drug-Resistant Breast Cancer. Ann. Biomed. Eng..

[10] Kučuk N., Primožič M., Knez Ž., Leitgeb M. (2023). Sustainable Biodegradable Biopolymer-Based Nanoparticles for Healthcare Applications. Int. J. Mol. Sci..

[11] Yi Z., Ye J., Kikugawa N., Kako T., Ouyang S., Stuart-Williams H., Yang H., Cao J., Luo W., Li Z. (2010). An Orthophosphate Semiconductor with Photooxidation Properties under Visible-Light Irradiation. Nat. Mater..

[12] Paluch E., Seniuk A., Plesh G., Widelski J., Szymański D., Wiglusz R.J., Motola M., Dworniczek E. (2023). Mechanism of Action and Efficiency of Ag_3_PO_4_-Based Photocatalysts for the Control of Hazardous Gram-Positive Pathogens. Int. J. Mol. Sci..

[13] Steckiewicz K.P., Zwara J., Jaskiewicz M., Kowalski S., Kamysz W., Zaleska-Medynska A., Inkielewicz-Stepniak I. (2019). Shape-Depended Biological Properties of Ag_3_PO_4_ Microparticles: Evaluation of Antimicrobial Properties and Cytotoxicity in In Vitro Model—Safety Assessment of Potential Clinical Usage. Oxid. Med. Cell. Longev..

[14] Ghavidel N., Fatehi P. (2021). Recent Developments in the Formulation and Use of Polymers and Particles of Plant-Based Origin for Emulsion Stabilizations. ChemSusChem.

[15] Al-Jubori Y., Ahmed N.T.B., Albusaidi R., Madden J., Das S., Sirasanagandla S.R. (2023). The Efficacy of Gum Arabic in Managing Diseases: A Systematic Review of Evidence-Based Clinical Trials. Biomolecules.

[16] Iravani S. (2020). Plant Gums for Sustainable and Eco-Friendly Synthesis of Nanoparticles: Recent Advances. Inorg. Nano-Metal Chem..

[17] Mhinzi G.S. (2003). Intra-Species Variation of the Properties of Gum Exudates from *Acacia senegal* var. *senegal* and *Acacia seyal* var. *fistula* from Tanzania. Bull. Chem. Soc. Ethiop..

[18] Fatmasari N., Kurniawan Y.S., Jumina J., Anwar C., Priastomo Y., Pranowo H.D., Zulkarnain A.K., Sholikhah E.N. (2022). Synthesis and In Vitro Assay of Hydroxyxanthones as Antioxidant and Anticancer Agents. Sci. Rep..

[19] Venkatesan J., Hur W., Gupta P.K., Son S.E., Lee H.B., Lee S.J., Ha C.H., Cheon S.H., Kim D.H., Seong G.H. (2023). Gum Arabic-Mediated Liquid Exfoliation of Transition Metal Dichalcogenides as Photothermic Anti-Breast Cancer Candidates. Int. J. Biol. Macromol..

[20] Lopez-Torrez L., Nigen M., Williams P., Doco T., Sanchez C. (2015). *Acacia senegal* vs. *Acacia seyal* Gums—Part 1: Composition and Structure of Hyperbranched Plant Exudates. Food Hydrocoll..

[21] Almohaimeed H.M., Waly H., Abou Khalil N.S., Hassanein K.M.A., Alkhudhairy B.S.M., Abd-Allah E.A. (2022). Gum Arabic Nanoformulation Rescues Neuronal Lesions in Bromobenzene-Challenged Rats by Its Antioxidant, Anti-Apoptotic and Cytoprotective Potentials. Sci. Rep..

[22] Badisa R.B., Darling-Reed S.F., Joseph P., Cooperwood J.S., Latinwo L.M., Goodman C.B. (2009). Selective Cytotoxic Activities of Two Novel Synthetic Drugs on Human Breast Carcinoma MCF-7 Cells. Anticancer Res..

[23] Al-Assaf S., Lukanowski J., Tretzel J. (2019). Gum Arabic from Acacia Seyal. European Patent.

[24] Biswas P., Dey D., Biswas P.K., Rahaman T.I., Saha S., Parvez A., Khan D.A., Lily N.J., Saha K., Sohel M. (2022). A Comprehensive Analysis and Anti-Cancer Activities of Quercetin in ROS-Mediated Cancer and Cancer Stem Cells. Int. J. Mol. Sci..

[25] Ullah I., Khalil A.T., Ali M., Iqbal J., Ali W., Alarifi S., Shinwari Z.K. (2020). Green-Synthesized Silver Nanoparticles Induced Apoptotic Cell Death in MCF-7 Breast Cancer Cells by Generating Reactive Oxygen Species and Activating Caspase 3 and 9 Enzyme Activities. Oxid. Med. Cell. Longev..

[26] Trachootham D., Alexandre J., Huang P. (2009). Targeting Cancer Cells by ROS-Mediated Mechanisms: A Radical Therapeutic Approach?. Nat. Rev. Drug Discov..

[27] Aruoma O.I., Halliwell B., Hoey B.M., Butler J. (1989). The Antioxidant Action of N-Acetylcysteine: Its Reaction with Hydrogen Peroxide, Hydroxyl Radical, Superoxide, and Hypochlorous Acid. Free Radic. Biol. Med..

[28] Zulkifli N.I., Muhamad M., Mohamad Zain N.N., Tan W.-N., Yahaya N., Bustami Y., Abdul Aziz A., Nik Mohamed Kamal N.N.S. (2020). A Bottom-Up Synthesis Approach to Silver Nanoparticles Induces Anti-Proliferative and Apoptotic Activities Against MCF-7, MCF-7/TAMR-1 and MCF-10A Human Breast Cell Lines. Molecules.

[29] Hamid A. (2017). Cytotoxic and Genotoxic Effects of Zerumbone on Wehi 7.2 Wild Type Murine Thymoma Cells. J. Agric. Sci..

[30] Rauf A., Imran M., Khan I.A., Ur-Rehman M., Gilani S.A., Mehmood Z., Mubarak M.S. (2018). Anticancer Potential of Quercetin: A Comprehensive Review. Phytother. Res..

[31] Murtaza M., Tariq Z., Kamal M.S., Rana A., Saleh T.A., Mahmoud M., Alarifi S.A., Syed N.A. (2024). Improving Water-Based Drilling Mud Performance Using Biopolymer Gum: Integrating Experimental and Machine Learning Techniques. Molecules.

[32] Mutharani B., Keerthi M., Chen S.-M., Ranganathan P., Chen T.-W., Lee S.-Y., Chang W.-H. (2020). One-Pot Sustainable Synthesis of Ce2S3/Gum Arabic Carbon Flower Nanocomposites for the Detection of Insecticide Imidacloprid. ACS Appl. Mater. Interfaces.

[33] Hassani A., Azarian M.M.S., Ibrahim W.N., Hussain S.A. (2020). Preparation, Characterization and Therapeutic Properties of Gum Arabic-Stabilized Gallic Acid Nanoparticles. Sci. Rep..

[34] Al-Ansari M.M., Al-Dahmash N.D., Ranjitsingh A.J.A. (2021). Synthesis of Silver Nanoparticles Using Gum Arabic: Evaluation of Its Inhibitory Action on *Streptococcus mutans* Causing Dental Caries and Endocarditis. J. Infect. Public Health.

[35] Alzahrani E. (2017). Colorimetric Detection Based on Localised Surface Plasmon Resonance Optical Characteristics for the Detection of Hydrogen Peroxide Using Acacia Gum–Stabilised Silver Nanoparticles. Anal. Chem. Insights.

[36] Kabbashi N., Alawdat T.M., Qudsieh I.Y., Alam M.Z., Shahabuddin M. (2022). Extraction of Bioactive Compound from Acacia Seyal Gum, in Vitro Evaluation of Antitumor Activity of Its Crude Extract against Leukemia. Int. J. Plant Based Pharm..

[37] Bhagya P.M., Rani T.S., Krishna T.P.A. (2026). Anticancer Potential of Nano-Engineered Silver Nanoparticle from Acacia Arabica Aqueous Seed Extract against a Prostate PC-3 Cancer Cell Line. Chem. Pap..

[38] Hassani A., Mahmood S., Enezei H.H., Hussain S.A., Hamad H.A., Aldoghachi A.F., Hagar A., Doolaanea A.A., Ibrahim W.N. (2020). Formulation, Characterization and Biological Activity Screening of Sodium Alginate-Gum Arabic Nanoparticles Loaded with Curcumin. Molecules.

[39] Ye A., Flanagan J., Singh H. (2006). Formation of Stable Nanoparticles via Electrostatic Complexation between Sodium Caseinate and Gum Arabic. Biopolymers.

[40] Thakkar A.B., Subramanian R.B., Thakkar V.R., Bhatt S.V., Chaki S., Vaidya Y.H., Patel V., Thakor P. (2024). Apoptosis Induction Capability of Silver Nanoparticles Capped with *Acorus calamus* L. and *Dalbergia sissoo* Roxb. Ex DC. against Lung Carcinoma Cells. Heliyon.

[41] Safari M., Naseri M., Naderi E., Esmaeili E. (2022). Magnetically Targeted Delivery of Quercetin-Loaded Ca1–xMnxFe2O4 Nanocarriers: Synthesis, Characterization and In Vitro Study on HEK 293-T and MCF-7 Cell Lines. Appl. Phys. A.

[42] Mosmann T. (1983). Rapid Colorimetric Assay for Cellular Growth and Survival: Application to Proliferation and Cytotoxicity Assays. J. Immunol. Methods.

[43] Kig C., Mertoglu E., Caliskan A., Hincal Agus H., Onay Ucar E., Guler V. (2021). Selective and Oxidative Stress-Mediated Cell Death of MCF-7 Cell Line Induced by Terpinolene. Biologia.

[44] Bézivin C., Tomasi S., Lohézic-Le Dévéhat F., Boustie J. (2003). Cytotoxic Activity of Some Lichen Extracts on Murine and Human Cancer Cell Lines. Phytomedicine.

[45] Elmore S. (2007). Apoptosis: A Review of Programmed Cell Death. Toxicol. Pathol..

[46] Vermes I., Haanen C., Steffens-Nakken H., Reutelingsperger C. (1995). A Novel Assay for Apoptosis. Flow Cytometric Detection of Phosphatidylserine Expression on Early Apoptotic Cells Using Fluorescein Labelled Annexin V. J. Immunol. Methods.

[47] Murakami A., Ashida H., Terao J. (2008). Multitargeted Cancer Prevention by Quercetin. Cancer Lett..

